# Timing and outcomes after silicone oil removal in proliferative vitreoretinopathy: a retrospective clinical series

**DOI:** 10.1186/s40942-015-0002-y

**Published:** 2015-04-15

**Authors:** Renata Leite De Pinho Tavares, Mário Junqueira Nóbrega, Fernando Amaral Junqueira Nóbrega, Fernando José De Novelli, Carlos Augusto Cardim De Oliveira

**Affiliations:** 1 Hospital de Olhos de Londrina (HOFTALON), Londrina, PR - BrazilRua Belo Horizonte 1330, ap 1401, Londrina, PR CEP 86020-060 Brazil; 2 Hospital de Olhos Sadalla, Universidade da Região de Joinville, Amin Ghanem, Joinville, SC Brazil; 3 Universidade de Alfenas (UNIFENAS), Alfenas, MG Brazil

**Keywords:** Retinal detachment, Silicone oil, Proliferative vitreoretinopathy

## Abstract

**Objective:**

To evaluate anatomical and functional outcomes after silicone oil extraction in patients with retinal detachment and proliferative vitreoretinopathy in an eye care referral center in Joinville, SC, southern Brazil.

**Methods:**

Retrospective, noncomparative study of patients with retinal detachment and posterior proliferative vitreoretinopathy followed up after silicone oil removal. Prophylactic 360-degree peripheral laser photocoagulation was performed one to three months before silicone oil extraction. Patients with cataract underwent a combined clear corneal phacoemulsification with intraocular lens implantation in the same procedure. Anatomical outcomes were related to the duration of silicone oil tamponade and the surgical procedure performed. Functional outcomes were divided into three categories (stability, worsening, or improvement) according to visual acuity variation before the surgery and at the last follow-up visit.

**Results:**

Fifty-three patients were followed up for a mean period of 1,262 days.

Fourteen eyes (26.4%) underwent cataract surgery combined with silicone oil extraction. Forty-eight eyes (90.5%) had attached retina at the last follow-up examination. Time of intraocular tamponade and association of phacoemulsification with silicone oil extraction were not considered as risk factors for retinal redetachment. Twenty-three cases (43.4%) showed visual acuity improvement, whereas 11 cases (20.8%) were stable and 19 cases (35.8%) showed visual acuity worsening. Five patients with attached retina had unexplained optic disc atrophy.

**Conclusion:**

Most patients had good anatomical and visual outcomes after silicone oil extraction. Prophylactic 360-degree laser retinopexy may have led to favorable outcomes. Benefits of silicone oil extraction and the associated risks of complications due to a new surgical procedure must be carefully evaluated before surgical indication.

## Background

Proliferative vitreoretinopathy (PVR) is an important complication of rhegmatogenous retinal detachment and its treatment requires a long-acting endotamponade, such as silicone oil, to reduce the rate of recurrent retinal detachment [1,2].

To avoid long-term complications due to the presence of silicone oil inside the eye, such as cataract, glaucoma and ceratopathy, its removal is usually necessary. Nevertheless, after silicone oil extraction, recurrence of PVR and consequently retinal redetachment can occur. It is generally associated with residual vitreoretinal traction at the vitreous base [3,4]; some other factors may also contribute to unfavorable outcomes, like intra and postoperative inflammation, intraoperative bleeding, retinal pigment epithelium exposure, retinectomy and extended duration of the surgical procedure.

As suggested by some authors, a prophylactic 360-degree laser retinopexy prior to silicone oil extraction may help to reduce retinal redetachment rates [5-7]. Additionally, in spite of a permanent attached retina, some patients may have unexplained vision loss after silicone oil extraction [1,3-7].

The objective of the present study is to evaluate anatomical and functional outcomes after silicone oil removal in patients with retinal detachment and proliferative vitreoretinopathy in an eye care referral center in Joinville, SC, southern Brazil.

## Methods

A retrospective, noncomparative clinical series of patients with rhegmatogenous retinal detachment and posterior PVR grade A, B, C1, C2, C3, D1 and D2 examined after silicone oil extraction. Surgical interventions were performed between January 1997 and February 2013. The patients were examined and operated on by the same surgeon (MJN).

**Figure 1:**
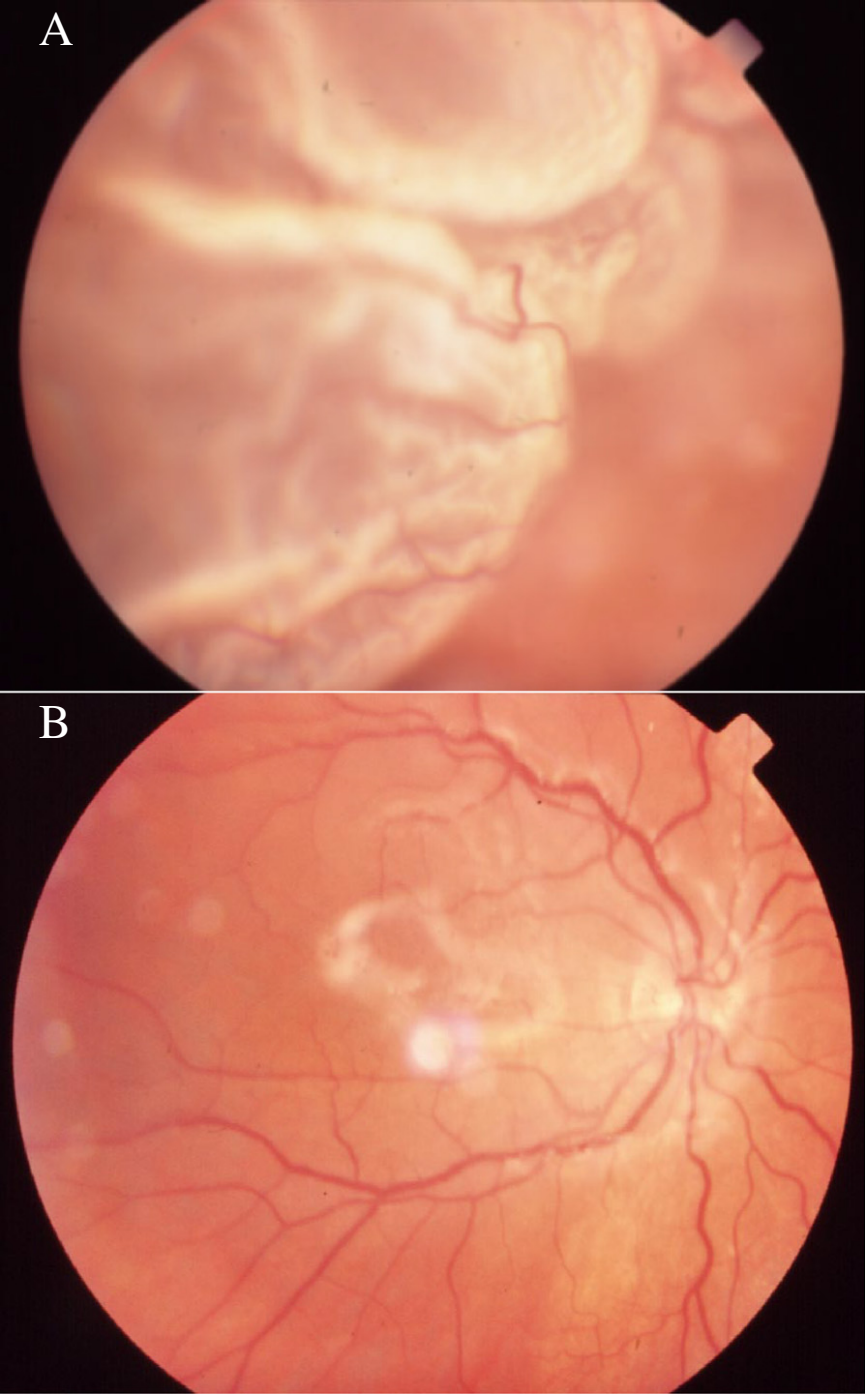
**Inicial operative procedure: rhegmatogenous retinal detachment and grade C posterior proliferative vitreoretinopathy (A) and postoperative result after silicone oil infusion (B).**

The initial operative procedure consisted of 20-gauge pars plana vitrectomy, 360-degree scleral buckle, laser endophotocoagulation surrounding retinal tears, and infusion of 5000-centistoke silicone oil (Ophthalmos®, São Paulo, SP) into the vitreous cavity (Figure 1). Prophylactic 360-degree peripheral laser photocoagulation was performed one to three months before silicone oil extraction. Laser spots were delivered to the scleral buckle area in two or three rows via slit lamp.

Silicone oil was extracted between 6 weeks and 12 months after the initial procedure through a standard two-port pars plana surgery. If there was cataract, a combined clear corneal incision phacoemulsification and intraocular lens (IOL) implantation was performed. During follow-up period, logMAR best-corrected visual acuity (BCVA), intraocular pressure (IOP), slit-lamp biomicroscopy, indirect and slit-lamp ophthalmoscopy were registered.

Functional outcomes were divided into three categories. Patients who had a final logMAR BCVA showing a 0.1-variation compared to the baseline BCVA were considered stabilized; a visual acuity variation was significant if the difference between baseline and final BCVA was 0.2 logMAR. A severe visual worsening was registered if there was a BCVA reduction of 0.3 logMAR or more.

Statistical analysis was performed using STATA version 12.0 (Texas USA). Categorical variables were expressed by frequencies and percentages and the continuous variables were expressed through means and standard deviation, with a confidence interval of 95%. The Student’s t-test was used to evaluate relation between mean period of silicone oil tamponade and retinal detachment as well as to compare visual acuity means before and after silicone oil removal. Pearson’s Chi-square test, or the Fischer exact test, was used to assess causality of using the combined procedure (silicone oil removal, phacoemulsification and IOL implantation) with retinal re-detachment cases, and also correlate it with final visual acuity. Significance level of 5% was accepted to reject the null hypothesis.

The informed consent to participate in the study was obtained from each patient. Hospital Municipal São José’s ethical committee, Joinville – Santa Catarina, Brazil, approved the research according to the project number: 240.558.

## Results

**Figure 2:**
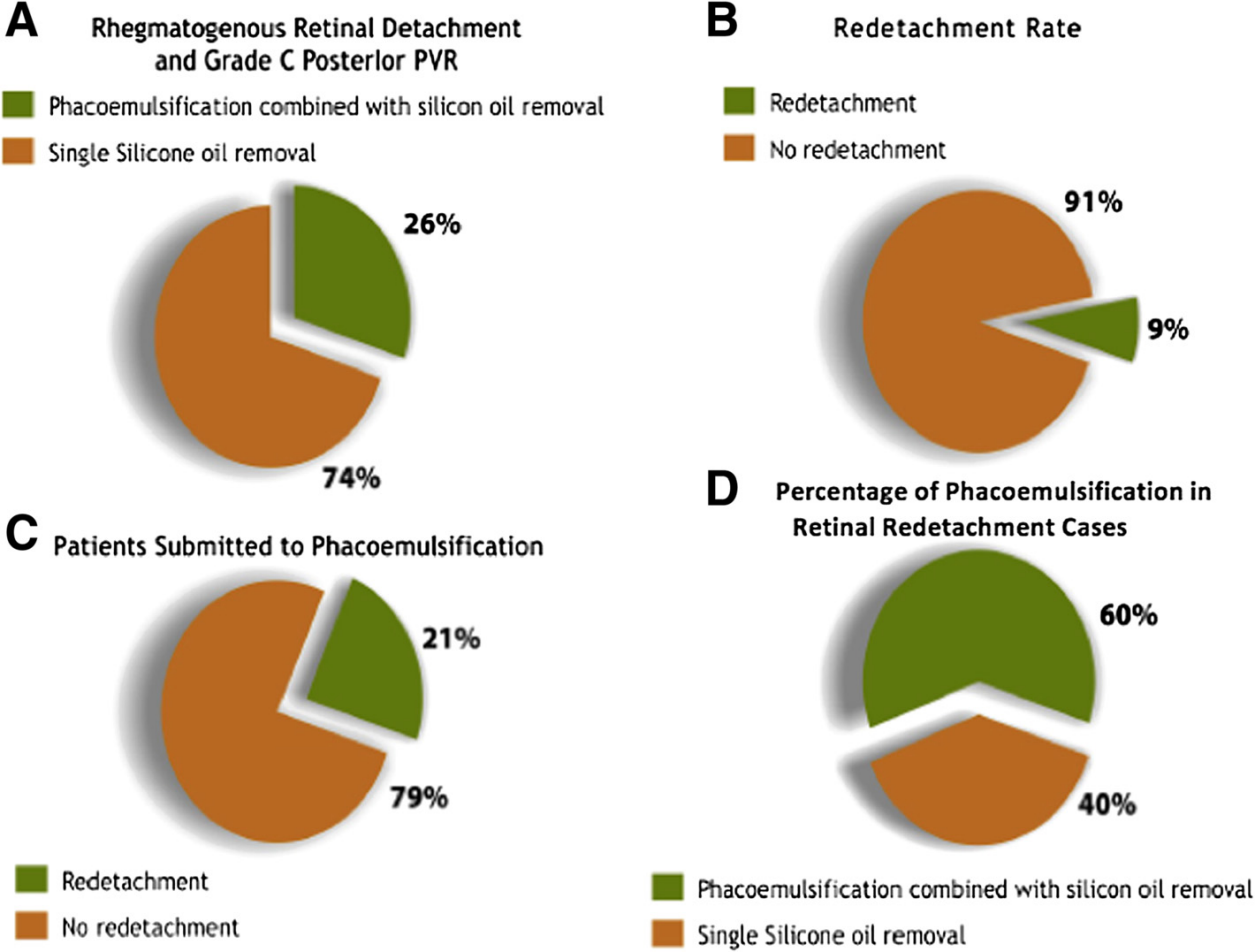
**Anatomical results after silicone oil removal and comparison between single and combined procedure with phacoemulsification.**

Fifty-three eyes of 53 patients were included; thirty-three (62%) patients were male; mean age was 52.7 years (range 16–79 years). According to PVR classification, 3.8% (2/53) of the patients were grade A PVR; 5.6% (3/53) grade B PVR; 32.1% (17/53) grade C1 PVR; 18.9% (10/53) grade C2 PVR; 5.6% (3/53) grade C3 PVR; 1.9% (1/53) grade D1 PVR and 1.9% (1/53) grade D2 PVR; 30.2% (16/53) of the patients had no classifying register. Follow-up varied from 256 days to 4,421 days (mean 1,262 + −944 days). The median time of silicone oil extraction was 196 days after its placement. The mean intraocular pressure was 15.6 + −7.4 before and 14.8 + −6.2 after silicone oil removal. Phacoemulsification with IOL implantation was combined with silicone oil removal in 14 eyes (26.4%) (Figure 2A).

Forty-eight (90.5%) patients had attached retina at the last follow-up visit (Figure 2B). The mean period of silicone oil duration in the vitreous cavity was 228 + −152 and 275 + −265 days in cases with and without redetachment, respectively. There was no association between duration of intraocular silicone oil and risk for retinal redetachment (p = 0.6997). The rate of retinal redetachment in patients submitted to phacoemulsification combined with silicone oil extraction was 21.4% (Figure 2C), which represented 60% of all the redetachment cases (Figure 2D), but not statiscally significant (p = 0.0735).

**Figure 3:**
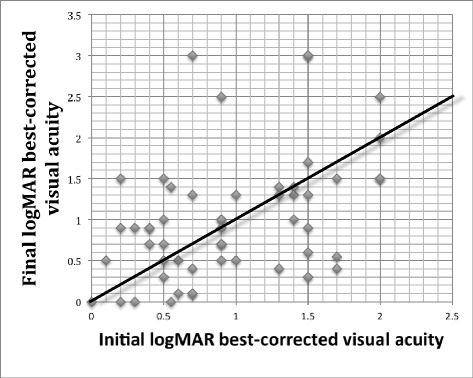
**Comparison of BCVA between preoperative examination and last follow-up assessment.**

Twenty-three patients (43.4%) showed BCVA improvement, eleven patients (20.8%) were stabilized and 19 patients (35.8%) had a BVCA worsening. Mean preoperative and postoperative values of BCVA were 0.95 logMAR (Snellen = 20/160) and 1.01 logMAR (Snellen = 20/200) respectively (Figure 3). Severe visual worsening was observed in 15 patients (28.3%), of whom five had unexplained optic disc atrophy.

Combined phacoemulsification with intraocular lens implant and removal of silicone oil did not influence the final visual acuity when compared to the isolated extraction of silicone oil (p = 0.426). In addition, BCVA worsening could not be directly associated with silicone oil removal (p = 0.6598).

## Discussion

Several factors may be related to anatomic and functional outcomes after silicone oil extraction in PVR cases. In this study, the relation between duration of silicone oil tamponade and incidence of retinal redetachment was not established. In the literature, while some authors did not consider the timing of silicone oil removal as a risk factor for anatomic success rate [4,8,9], others observed that shorter tamponade duration had lower attachment rate than longer tamponade duration [10,11].

The association of encircling buckle and peripheral laser photocoagulation prior to silicone oil extraction has been previously reported as safe and advantageous [9,12-15]. A 360-degrees laser performed previously to surgery may improve peripheral chorioretinal adhesion and avoid retinal redetachment in spite of residual vitreous base traction [3,7,16].

Compared to other studies (Table 1), the present report showed a low redetachment rate (9.5%) after silicone oil extraction. This may be due to improved surgical management of complicated retinal detachments in the last years, especially adequate vitreous removal with the use of wide-field viewing systems [7,12].

**Table 1:** **Previous studies showing rate of retinal redetachment after silicone oil extraction and number of treated eyes**

	**% Redetach**	**Treat eyes**
Bassat et al. [22] (2000)	9.3	32
Flaxel et al. [23] (2000)	34	62
Scholda et al. [11] (2000)	16.1	112
Assi et al. [24] (2001)	20.2	74
Falkner et al. [1] (2001)	16.5	103
Jonas et al. [4] (2001)	27.6	185
Jiang et al. [25] (2002)	20.2	168
Unlu et al. [26] (2004)	9.5	21
Scott et al. [27] (2005)	21	57
Soheilian et al. [28] (2006)	28	82
Lam et al. [8] (2008)	18.4	147
Jain et al. [15] (2010)	11.6	300
Nagpal et al. [9] (2012)	12.7	370

A combined surgery, with longer duration and more intense inflammation, may predispose to postoperative PVR and retinal redetachment [2]. The present study showed a higher rate of retinal detachment recurrence in eyes submitted to silicone oil extraction associated with cataract surgery than eyes that had only silicone oil removal (21.4% vs. 5.1%); however, this result was not statistically significant (p = 0.0735).

Functional outcomes were not fully correlated with anatomical outcomes. To date, there is no definite explanation for vision loss or optic atrophy after silicone oil extraction. A long-term and close contact of silicone oil with the retina may cause toxicity; abnormal potassium exchanges and dysfunction of central Müller cells may also explain a shallow foveal depression and vision loss [13-15].

Besides surgical intervention, new types of treatment for PVR are being tested in clinical trials. Several studies have been performed, including use of many drugs, such as daunorubicin, corticosteroids, 5-fluorouracil, heparin and colchicine [17-21]. Nonetheless, PVR is still a challenge and continues to be the most common cause of surgical failure following retinal detachment surgery.

The main limitation of the present study is its retrospective and noncomparative design. Because of the patients’ records were not standardized, some complementary data, such as analysis of macular optic coherence tomography and visual field, could not be included. The size of the sample may also have influenced the results; a higher number of treated eyes could permit other comparisons and demonstrate significant differences between the outcomes.

As described in other reports, this study shows how critical is the dilemma concerning treatment of complex rhegmatogenous retinal detachment. Silicone oil removal provided favorable anatomical and functional results in the majority of the operated eyes and prophylactic peripheral laser retinopexy may have contributed to this. However, some eyes developed unexplained vision loss after silicone oil extraction and there was also a trend to retinal redetachment when silicone oil removal was combined with phacoemulsification and intraocular lens placement.

## Conclusion

In summary, low detachment rate may be due to improved surgical management of complicated retinal detachments and also to prophylactic 360- degree retinopexy. Surgeons must be aware of adverse results associated with silicone oil removal. Benefits of silicone oil extraction must be outweighed against its extended duration in the eye and the possibility of complications after a new surgical procedure.

## Abbreviations

PVR: Proliferative vitreoretinopathy
IOL: Intraocular lens
BCVA: Best-corrected visual acuity
